# Supplementary figures and images for: Could the new coronavirus have infected humans prior November 2019?
Source: PLoS One. 2021 Aug 19;16(8):e0248255. doi: 10.1371/journal.pone.0248255 (PMC8375974; doi:10.1371/journal.pone.0248255)

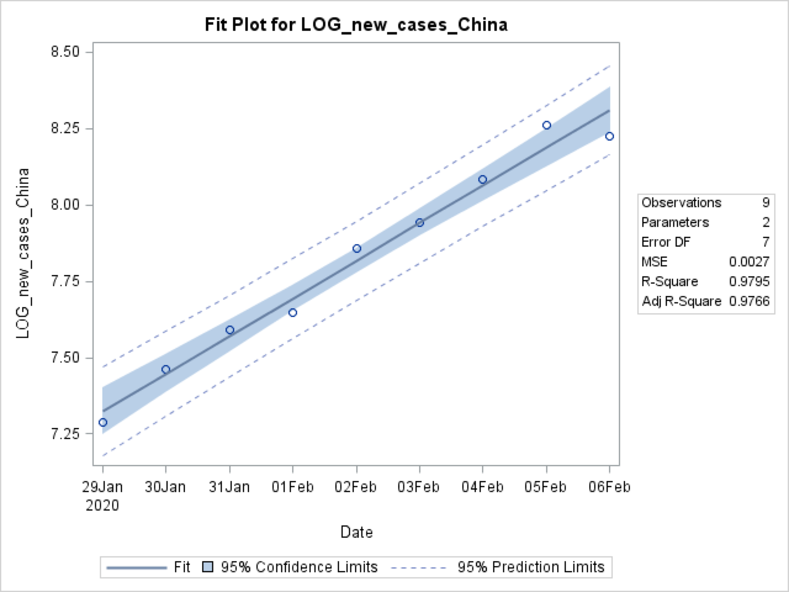

Supplement: S1 Fig — (TIF) [file pone.0248255.s002.tif]

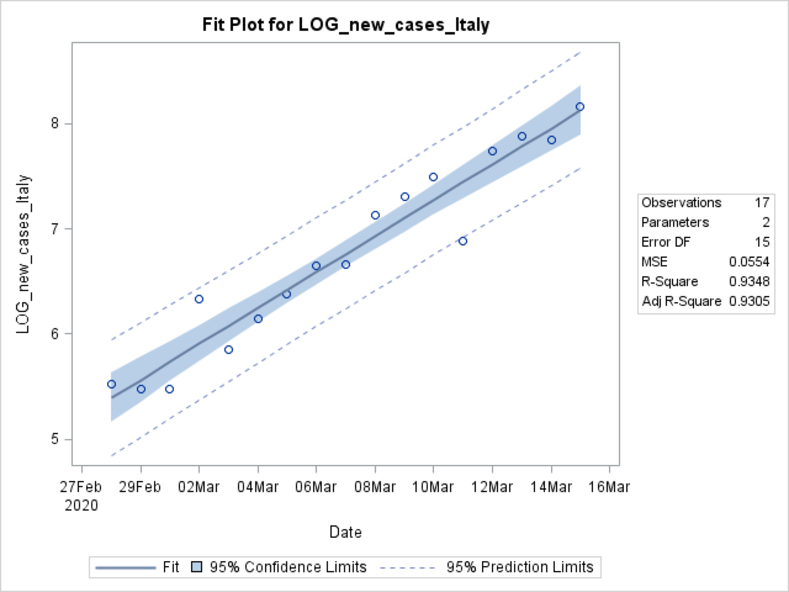

Supplement: S2 Fig — (TIF) [file pone.0248255.s003.tif]

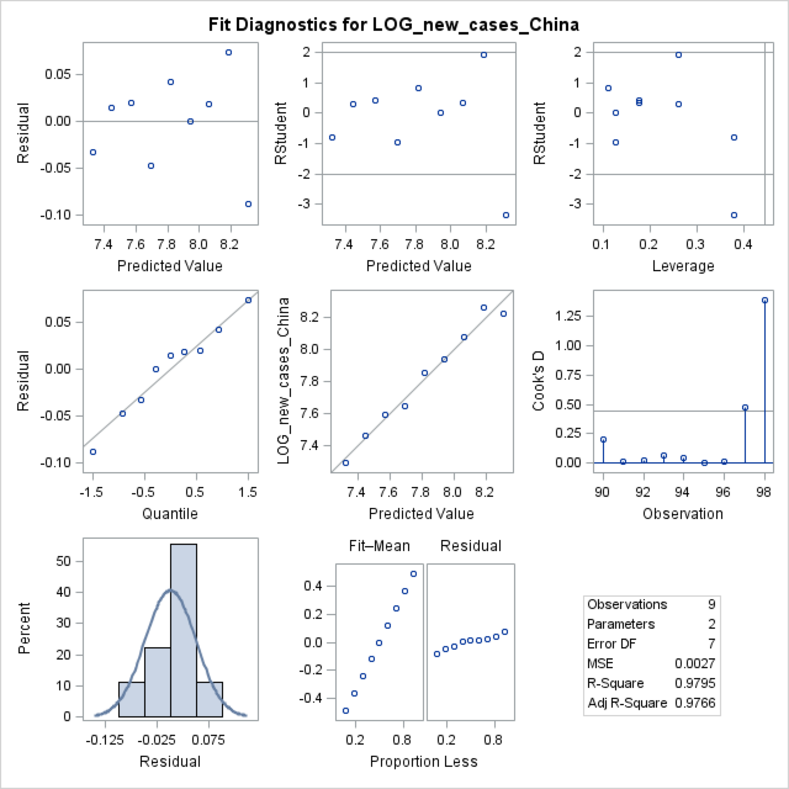

Supplement: S3 Fig — (TIF) [file pone.0248255.s004.tif]

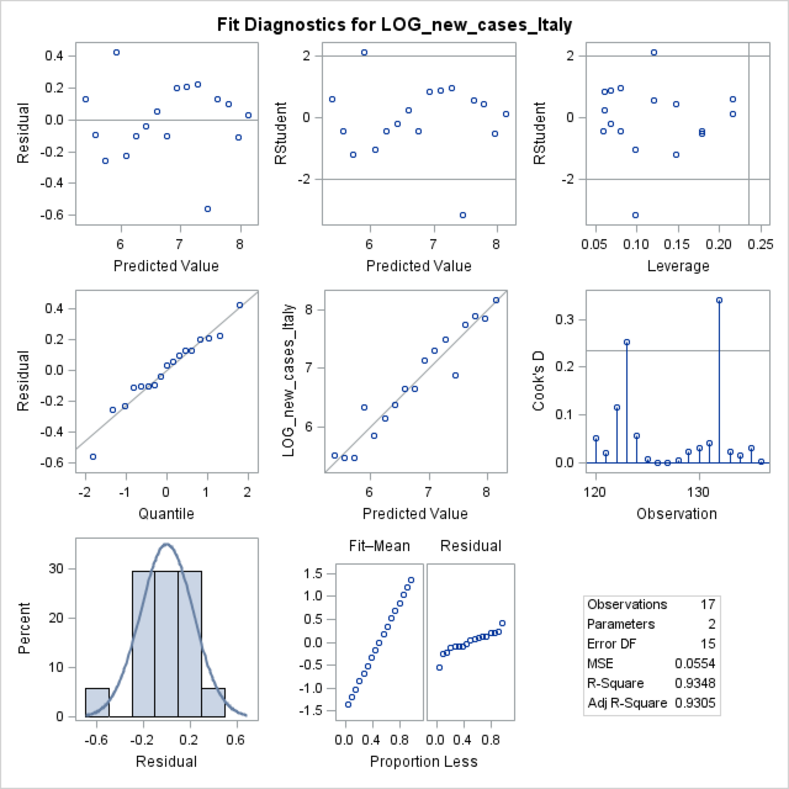

Supplement: S4 Fig — (TIF) [file pone.0248255.s005.tif]
